# Association Between the Alteration of Acoustic Reflex and Central Auditory Processing Disorder in Children and Adolescents: A Systematic Review

**DOI:** 10.1055/a-2804-5151

**Published:** 2026-10-23

**Authors:** Elora Bion Pereira, Georgea Espindola Ribeiro, Daniela Polo Camargo da Silva

**Affiliations:** 1 Department of Speech-Language Pathology and Audiology, Universidade Federal de Santa Catarina (UFSC), Florianópolis, SC, Brazil; 2 Hospital das Clínicas, Botucatu Medical School (HCFMB), School of Medicine of Botucatu, Universidade Estadual Paulista (UNESP), Botucatu, SP, Brazil

**Keywords:** reflex, acoustic, hearing tests, auditory perception, auditory diseases, central, systematic review

## Abstract

**Introduction:**

Studies have investigated the relationship between acoustic reflex (AR) abnormalities and central auditory processing disorder (CAPD). Evidence suggests that the absence or alteration of AR may be associated with CAPD, indicating possible involvement of central auditory pathways.

**Objective:**

To systematically review the literature on the association between AR abnormalities and CAPD in children and adolescents.

**Data Synthesis:**

This systematic review was registered in PROSPERO (CRD42023440214) and conducted according to the Preferred Reporting Items for Systematic Reviews and Meta-Analyses (PRISMA) guidelines. Searches were performed in PubMed/MEDLINE, Scopus, Web of Science, Embase, LILACS, LIVIVO, ProQuest and Google Scholar, without language or date restrictions. Observational studies addressing AR and CAPD in children and adolescents were included. Exclusion criteria comprised other study designs, non-traditional AR measurements, inclusion of adults or elderly participants, CAPD associated with other conditions, analysis limited to AR presence, lack of AR–CAPD comparison, absence of age reporting, or inaccessibility of the full text. Data regarding study characteristics, participants, exposure, and outcomes were extracted. Three reviewers independently screened studies, while two performed data extraction and quality assessment. Disagreements were resolved by consensus. From 2,333 identified records, six studies met the inclusion criteria. Four reported a higher prevalence of AR abnormalities in children with CAPD. Most studies presented moderate methodological quality.

**Conclusions:**

Altered ARs are more frequently observed in children with CAPD; however, the available evidence is limited and heterogeneous. Current findings do not support AR as a definitive diagnostic marker for CAPD. Further research integrating behavioral and electrophysiological measures is necessary to clarify its clinical utility.

## Introduction

Central auditory processing (CAP) refers to a set of neurophysiological processes that allow listeners to efficiently interpret auditory information. 1 2 These skills include sound lateralization and localization, auditory discrimination, pattern and temporal recognition, as well as temporal integration and masking. Additionally, CAP encompasses the ability to process competing and degraded acoustic signals, which are essential for auditory comprehension in complex environments. 1 2

Central auditory processing disorder (CAPD) is characterized by deficits in processing auditory signals that are not related to hearing loss or cognitive impairment, but affect one or more auditory skills. 3 4 These deficits may compromise an individual's ability to attend to, discriminate, recognize, and understand auditory information, leading to difficulties in comprehension, behavioral changes, and academic challenges. 3 4

The development of CAP depends on several factors, including auditory experiences, the integrity of the auditory system, and brain activity. 5 Efficient CAP function requires proper functioning of the peripheral auditory structures to ensure accurate transmission of acoustic information to the central nervous system. 5 6

Among the auditory mechanisms that contribute to the proper transmission of sound stimuli, the acoustic reflex (AR) of the stapedius muscle plays a significant role. The AR contributes to sound localization, auditory attention, speech detection and recognition, background noise reduction, and inner ear protection. 7 AR testing involves presenting high-intensity acoustic stimuli that trigger contraction of the stapedius muscle, and its integrity depends on tympano-ossicular function and both afferent and efferent auditory pathways. 8 9

The neural circuit of the reflex arc is mediated by brainstem structures, particularly the superior olivary complex, which is also essential for CAP mechanisms. Previous studies have investigated the relationship between AR abnormalities and CAPD, suggesting a possible association between them. 10 11 12

Given this context, the objective of this systematic review was to investigate the association between AR abnormalities and the occurrence of CAPD in children and adolescents, contributing to evidence-based clinical practice in pediatric audiology.

## Review of Literature

### Research Strategy

This review was conducted following the protocol registered in PROSPERO (CRD42023440214) and reported following the Preferred Reporting Items for Systematic Reviews and Meta-Analyses (PRISMA) 2020 guidelines. 13

Observational studies conducted in children and adolescents that aimed to evaluate the association between AR abnormalities and the occurrence of CAPD were included.

The following studies were excluded: studies evaluating sensitized AR; sample including individuals with autism spectrum disorder; studies that did not perform CAP assessment; sample including only participants with present AR; studies for which the full article/thesis was not found; studies with no comparison between AR and CAPD results; sample including adults; studies with lack of age reporting; conference abstracts, editorials, and review articles; and studies that performed only one CAP test.

The search was conducted on October 18, 2023, in the following databases, including gray literature: PubMed/MEDLINE, LILACS, Scopus, Web of Science, Embase, ProQuest, LIVIVO, and Google Scholar, with no restrictions on language or year of publication. To ensure methodological rigor, forward and backward citation tracking was performed using CitationChaser. 14

The research question was structured using the PECOS framework (Patient, Exposure, Comparison, Outcomes, Study Design): “In children and adolescents, is there an association between abnormal AR and the occurrence of CAPD?” ( Table 1 ).

**Table 1:** Key question formulated using the PECOS strategy

**“** In children and adolescents, is there an association between AR alterations and the occurrence of CAPD?”	
**P**	Children and adolescents
**E**	Acoustic reflex alteration
**C**	Presence of the acoustic reflex
**O**	Central auditory processing disorder
**S**	Observational studies

Abbreviations: AR, acoustic reflex; CAPD, central auditory processing disorder; PECOS, Population, Exposure, Comparator, Outcome, Study design.

Search descriptors were selected from MeSH and DeCS, in both English and Portuguese, and combined using “AND” and “OR” Boolean operators ( Appendix 1 ).

### Selection Criteria

The selection process was conducted in two phases. In phase one, three independent reviewers screened titles and abstracts. In phase two, full-text screening was conducted independently by the same reviewers. Conflicts were resolved in consensus meetings.

Reference management software (EndNote Web® and Rayyan - Intelligent Systematic Review) was used to organize records, remove duplicates, and maintain a blinded reviewer screening process.

### Data Analysis

Two independent reviewers extracted the following data using standardized electronic forms: study characteristics (author, year, country, design); population characteristics (sample size, age, group description); exposure characteristics (AR results and CAP behavioral assessment); and outcome data (association between AR and CAPD).

Disagreements were resolved through discussion; when necessary, a third reviewer adjudicated. Authors were contacted up to three times (one-week intervals) to obtain missing data. Studies with unavailable data after attempts were excluded ( Appendix 2 ).

Methodological quality was assessed independently by two reviewers using the Joanna Briggs Institute (JBI) Critical Appraisal Checklist for Analytical Cross-Sectional Studies. 15 Disagreements were resolved by consensus. When reporting was insufficient, the risk of bias was classified as unclear. 15

Methodological quality was categorized as low (≤3 “yes”), moderate (4–6 “yes”) (4–6 “yes”) or high (≥7 “yes”). 16

## Results

A total of 2,333 articles were retrieved from database and gray literature searches. After removal of 623 duplicates, 1,710 records were screened by title and abstract (Phase 1). Of these, 22 studies were selected for full-text review (Phase 2), and 15 were excluded, resulting in five eligible articles. One additional study was identified through citation tracking via CitationChaser, 14 bringing the total to six studies included in the qualitative synthesis. The selection and exclusion processes are presented in Fig. 1 , and excluded studies from Phase 2, with justification, are listed in Appendix 2 .

**Fig. 1.**
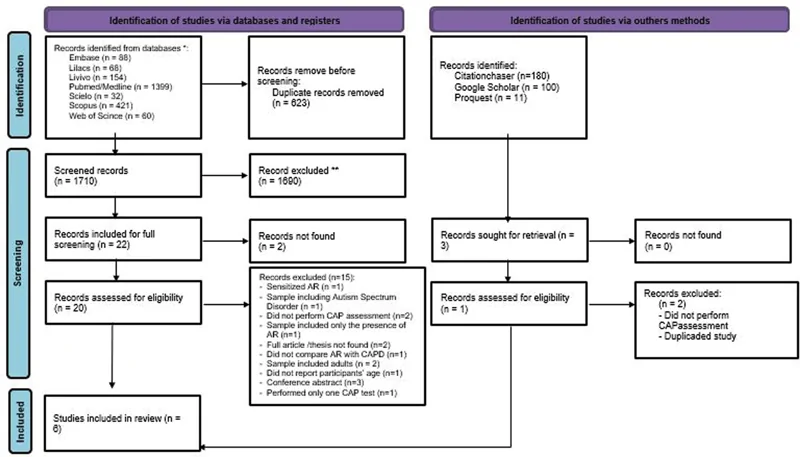
Flowchart of research criteria and literature selection. Adapted from *:* Page MJ, McKenzie JE, Bossuyt PM, et al. The PRISMA 2020 statement: an updated guideline for reporting systematic reviews. BMJ 2021;372:n71.

The six studies included were conducted in four countries: Brazil (n = 2), 7 17 Canada (n = 2), 18 19 Germany (n = 1), 20 and New Zealand (n = 1). 21 The age of participants ranged from 5 to 18 years. The main characteristics, assessment procedures, and findings of the six included studies are presented in Table 2 .

Sample sizes varied considerably across studies. The smallest sample was from Attoni et al. (2010), 7 with 22 children in the experimental group, while the largest was reported by Meneguello et al. (2001), 17 including 68 participants with CAPD.

All studies employed a cross-sectional design, although data collection procedures and assessment protocols varied across research groups.

### Central Auditory Processing Assessment

The six included studies utilized a range of behavioral CAP tests. Allen & Allan (2014) 18 applied monotic and dichotic listening tests, including filtered speech (FS), words in ipsilateral competition (WIC), staggered spondaic word test (SSW), pitch pattern sequence (PPS), and auditory fusion test-revised (AFT-R). In contrast, Attoni et al. (2010) 7 conducted diotic, monotic, and dichotic listening tests, such as the simplified auditory processing assessment (SAPA), pediatric speech intelligibility test (PSI), speech in noise (SN), dichotic digits test (DDT), and SSW. Kunze et al. (2017) 20 employed speech tests in monotic and dichotic listening, along with temporal resolution tests. Meneguello et al. (2001) 17 conducted diotic, monotic, and dichotic listening tests, including SAPA, SN, non-verbal dichotic test (NVDT), DDT, and SSW. Saxena et al. (2015) applied speech tests in monotic and dichotic listening, as well as temporal resolution tests, including SSW, PPS, WIC, and gap detection. Finally, Smart et al. (2019) 21 performed speech tests in monotic and dichotic listening, in addition to sequencing and temporal resolution tests, such as frequency pattern test (FPT), DDT, compressed and reverberated words (CRW), gaps-in-noise (GIN), masking level difference (MLD), and speech in spatial noise (SSN).

### Acoustic Reflex Assessment

AR testing protocols also varied. In general, AR was evaluated in both groups of participants, those with and without CAPD, 7 18 19 21 whereas some studies analyzed only the CAPD group in relation to AR abnormalities. 17

While the majority of the studies assessed ipsilateral and contralateral reflex across multiple frequencies (commonly 500, 1000, 2000, and 4000 Hz), some studies evaluated specific frequencies only.

**Table 2:** Characteristics of the included studies (n = 6)

Author/year/country	Study design	Sample characteristics	Objective	Acoustic reflex results	CAP test results	Conclusion
Allen & Allan, 2014 Canada 18	Cross-sectional	CAPD group: 40 children (ages 7–17) Non-CAPD group: 23 children (ages 7–17).	To examine the relationship between clinical diagnosis of CAPD, based on behavioral tests and neural function measures including, AR and ABR	AR was measured at 500, 1000, and 2000 Hz, both ipsilateral and contralateral, with a maximum intensity of 110 dB. CAPD Group: 30 participants had present ARs both ipsilaterally and contralaterally. Non-CAPD Group: 16 participants had present ARs both ipsilaterally and contralaterally.	Tests assessing auditory abilities through monotic and dichotic listening were applied (FST, WIC, SSW, PPS, and AFT-R).	Abnormal responses were observed in both groups, including elevated thresholds and absent AR responses.
Attoni et al., 2010 Brazil 7	Cross-sectional	Experimental group: 22 children with phonological disorder (ages 5–7) Control group: 24 children (ages 5–7).	To investigate the relationship between auditory processing, acoustic reflex, and phonological disorders in children.	AR was assessed in both ears at 500, 1000, 2000, and 4000 Hz, both ipsilateral and contralateral. Experimental group: AR values outside normal limits or absent. Control group: AR values within normal limits.	Tests assessing auditory abilities through diotic, monotic, and dichotic listening were applied (SAPA, PSI, SN, DDT, and SSW).	All children with phonological disorder had abnormal AR.
Kunze et al., 2017 Germany 20	Cross-sectional	CAPD group: 57 children (mean age 9.5 years, range 6.3–13.2 years) Non-CAPD group: 50 children (mean age 9.7 years, range 8.8–10.4 years).	To determine the stapedial reflex thresholds in children with and without CAPD.	AR was assessed at 500, 1000, 2000, and 4000 Hz, both ipsilateral and contralateral, with a maximum intensity of 100 dB. No significant differences were found between groups regarding AR occurrence or threshold values.	Speech tests in monotic and dichotic listening and temporal resolution tests were applied.	The study results indicate a lack of clinical utility for AR in distinguishing children with and without CAPD.
Meneguello et al., 2001 Brazil 17	Cross-sectional	Study group: 68 individuals (ages 7–18).	To characterize AR measurements in patients with CAPD, seeking possible associations between AR abnormalities and CAPD.	AR was assessed at 500, 1000, 2000, and 4000 Hz, in the contralateral condition. AR was considered normal when present between 70-90 dB HL; results above 90 dB HL or absent were considered altered. Of the 97% of individuals with CAPD, 62% had AR abnormalities.	Tests assessing auditory abilities through diotic, monotic, and dichotic listening were applied (SAPA, SN, NVDT, DDT, and SSW).	AR was significantly altered in individuals with severe CAPD, in those with gnostic deficits in decoding (auditory analysis), encoding (auditory integration), and/or organization (auditory memory), and in those with multiple impaired auditory abilities.
Saxena et al., 2015 Canada 19	Cross-sectional	Study group: 37 children (ages 7–15) with academic impairment and difficulty understanding speech. Control group: 17 typical children (ages 7–15).	To investigate AR in typically developing children and those with CAPD.	AR was assessed at 500, 1000, and 2000 Hz, both ipsilateral and contralateral. Among the 37 children with academic impairment and difficulty understanding speech, 23 had CAPD. Of these, six had elevated AR thresholds, and four had absent AR. Among the remaining 14 children, seven had elevated AR thresholds, and three had absent AR. Among the 17 typical children, three had one or more elevated AR thresholds.	Speech tests in monotic and dichotic listening and temporal resolution tests were applied (SSW, PPS, WIC, and gap detection).	This study showed that children with CAPD frequently exhibit AR abnormalities.
Smart et al., 2019 New Zealand 21	Cross-sectional	Study group: 29 children with suspected CAPD (ages 7.3–12.9) Control group: 34 typically developing children (ages 7–12.1).	To determine whether children with auditory difficulties show objective evidence of efferent auditory function based on medial olivocochlear reflex and AR measurements.	AR was assessed at 1000 and 2000 Hz, both ipsilateral and contralateral. Study group children had significantly higher AR thresholds than the control group at 2000 Hz in the contralateral condition. A significantly higher proportion of study group participants had abnormal AR compared to the control group.	Speech tests in monotic and dichotic listening and ordering and temporal resolution tests were applied (FPT, DDT, CRW, GIN, MLD, and SSN).	AR results indicated signs of efferent system dysfunction in children with CAPD; however, this was not observed for the medial olivocochlear reflex.

**Abbreviations:** ABR, auditory brainstem response; AFT-R, auditory fusion test-revised; AR, acoustic reflex; CAPD, central auditory processing disorder; CRW, compressed and reverberated words; dB HL, decibel hearing level; DDT, dichotic digit test; FPT, frequency pattern test; FST, filtered speech test; GIN, gaps-in-noise; Hz, Hertz; MLD, masking level difference; NVDT, non-verbal dichotic test; PPS, pitch pattern sequence; PSI, pediatric speech intelligibility; SAPA, simplified auditory processing assessment; SN, speech in noise; SSN, speech in spatial noise; SSW, staggered spondaic word test; WIC, words in noise test.

Across studies, children and adolescents with CAPD demonstrated a higher prevalence of abnormal AR findings, including elevated thresholds or absent responses. 7 17 21 22 In contrast, Kunze et al. (2017) reported no significant AR threshold differences between CAPD and control groups.

Among the six included cross-sectional studies, only one article demonstrated high methodological quality. 21 The remaining studies presented moderate quality 7 17 18 19 20 ( Table 3 ).

**Table 3:** Results of the critical appraisal of methodological quality of included studies using the JBI tool for analytical cross-sectional studies

Studies	Q1	Q2	Q3	Q4	Q5	Q6	Q7	Q8	Total	Quality
Allen & Allan, 2014 18	Y	Y	Y	Y	U	U	Y	Y	6	Moderate
Attoni et al., 2010 7	Y	Y	Y	Y	U	U	Y	U	5	Moderate
Kunze et al., 2017 20	Y	Y	Y	Y	U	U	Y	Y	6	Moderate
Meneguello et al., 2001 17	Y	Y	Y	Y	N	U	Y	U	5	Moderate
Saxena et al., 2015 19	Y	Y	Y	Y	N	N	Y	Y	6	Moderate
Smart et al., 2019 21	Y	Y	Y	Y	Y	Y	Y	Y	8	High

**Abbreviations:** N, no; U, unclear; Y, yes.

**Q1.** Were the criteria for inclusion in the sample clearly defined? **Q2.** Were the study subjects and the setting described in detail? **Q3.** Was the exposure measured in a valid and reliable way? **Q4.** Were objective, standard criteria used for measurement of the condition? **Q5.** Were confounding factors identified? **Q6.** Were strategies to deal with confounding factors stated? **Q7.** Were the outcomes measured in a valid and reliable way? **Q8.** Was appropriate statistical analysis used?

**Note:** Methodological quality was categorized as “Low” when the study received up to three “Yes” responses for the evaluated items; “Moderate” when the study received four to six. “Yes” responses; and “High” when the study obtained seven or more “Yes” responses.

Among those with moderate quality, the identified limitations were attributed to the lack of identification of potential confounding factors in the sample and the absence of strategies to address these confounders within the investigated groups. 7 17 18 19 20

## Discussion

In this systematic review, six studies met the eligibility criteria and were included, two of which were conducted in Brazil. 7 17 This finding underscores the importance of Brazilian scientific contributions in the field of auditory disorders, highlighting a growing interest in this area of research. However, the findings reveal that, although there is a tendency toward an association between AR abnormalities and CAPD, this relationship is not consistent across the analyzed studies.

The CAPD classification is multifaceted, and as recommended by the American Speech-Language-Hearing Association (ASHA, 2006), 1 must be supported by a battery of behavioral tests assessing key auditory skills, including dichotic listening, low-redundancy monaural tests, auditory discrimination, binaural interaction, and temporal processing. 1 Across the included studies, a range of tests was applied, with SSW, DDT, and SAPA. 7 17 19 20 This variability in assessment protocols likely contributed to discrepancies in findings across studies.

The low-redundancy monaural tests are particularly important for detecting dysfunctions in brainstem regions such as the superior olivary complex, a structure also involved in AR pathways. These tests were applied in five out of the six studies. 7 17 18 19 20 Smart et al. (2019) 21 did not include this category, which may partially explain differences in outcomes.

Pediatric CAPD commonly co-occurs with other neurodevelopmental conditions, increasing heterogeneity. For instance, Attoni et al. (2010) 7 identified AR and CAP abnormalities in children with phonological disorders, demonstrating the challenges in isolating auditory-specific deficits. Given this complexity, comprehensive evaluation involving language, cognition, and efferent auditory function is essential to better characterize CAPD populations and reduce diagnostic overlap. Allen & Allan (2014) 18 exemplified this by including linguistic, cognitive, academic and electrophysiological measures in addition to AR testing.

Some studies reported the most common complaints among children and adolescents with CAPD, such as reading and writing difficulties, inattention, 17 and difficulty hearing in noise and understanding unfamiliar speech. 18

These symptoms align with the known functional role of the AR, which contributes to speech perception in noise by attenuating low-frequency energy and improving signal-to-noise ratio. 22 23

Consistent with this, Smart et al. (2019) 21 observed elevated AR thresholds at 2000 Hz in children with CAPD, particularly contralaterally, and a greater proportion of abnormal reflexes compared to controls (26% vs. 3%).

Similar patterns were found by Allen & Allan (2014) 18 and Meneguello et al. (2001), 17 who reported higher frequencies of absent or elevated AR thresholds in CAPD population. Saxena et al. (2015) 19 also reported flatter reflex growth functions in CAPD, particularly contralaterally.

In contrast, Kunze et al. (2017) 20 found no significant differences in AR thresholds between children with and without CAPD. These contrasting results highlight the inconsistency of the findings reported in the literature.

Differences in AR protocols likely contributed to result variability. For example, while Allen & Allan (2014) 18 and Saxena et al. (2015) 19 investigated AR responses at 500, 1000, and 2000 Hz, the studies by Attoni et al. (2010) 7 and Kunze et al. (2017) 20 also included 4000 Hz. In contrast, Smart et al. (2019) 21 restricted their assessment to 1000 and 2000 Hz. This variation in frequency selection may have influenced the detection of AR abnormalities.

Furthermore, not all studies evaluated both ipsilateral and contralateral reflexes. 7 18 19 20 21 Meneguello et al. (2001) 17 focused only on the contralateral condition.

The analysis revealed methodological heterogeneity among the included studies. Participants' ages ranged from 5 to 18 years, and AR assessment protocols differed in stimulus intensity (100-110 dB HL or unspecified). 20 23 There were also discrepancies in the values considered normal and in the criteria used to determine AR absence.

Collectively, these factors limit comparability and emphasize the need for standardized AR procedures in CAPD research.

Regarding the methodological quality, five of the six included cross-sectional studies demonstrated moderate quality, 7 17 18 19 20 and one demonstrated high quality 21 according to JBI criteria, indicating potential sources of systematic bias but not to the extent of invalidating the findings.

These observations underscore the need for future primary studies following minimum standards for CAP evaluation, including standardized behavioral batteries, careful consideration of middle-ear status, and pediatric samples aged ≥7 years to ensure test reliability.

Overall, current evidence regarding the association between AR abnormalities and CAPD remains limited, inconsistent, and fragmented. This review clarifies the existing evidence landscape and highlights the need for well-designed studies to determine whether AR can serve as a reliable clinical marker of CAPD.

## Final Comments

The heterogeneity of samples, assessment methods, and comorbidities limited the interpretation and generalization of the findings. Although abnormal ARs were more frequently observed in children and adolescents with CAPD, the current evidence remains insufficient and inconsistent to establish the AR as a standalone diagnostic marker.

The combination of CAP behavioral tests, AR assessment, and auditory evoked potentials may provide a more comprehensive and functional evaluation of CAPD, allowing for a better understanding of its clinical implications.
