# Supplementary material for: Association Between the Alteration of Acoustic Reflex and Central Auditory Processing Disorder in Children and Adolescents: A Systematic Review
Source: Int Arch Otorhinolaryngol. 2026 Oct 23;30(4):1–8. doi: 10.1055/a-2804-5151 (PMC13694147; doi:10.1055/a-2804-5151)
Supplement: Supplementary file 1 [file 10-1055-a-2804-5151-s251986-1.pdf]

Appendix 1. Search strategy for each eletrocnic database

Database	Search
PubMed/Medline	**("Auditory Perception"[Mesh] OR "Auditory Perception"** OR "Auditory Perceptions" OR "Auditory Processing" OR "Auditory behavior" OR "Auditory Behaviors" OR "Behavioral Auditory" OR **"Auditory Perceptual Disorders"[Mesh]** OR **"Auditory Perceptual Disorders"** OR "Auditory Perceptual Disorder" OR "Auditory Processing Disorder" OR "Auditory Processing Disorders" OR "Psychoacoustical Disorders" OR "Psychoacoustical Disorder" OR "Acoustic Perceptual Disorder" OR "Acoustic Perceptual Disorders" OR "Auditory Comprehension Disorder" OR "Auditory Comprehension Disorders" OR "Auditory Inattention" OR "Auditory Inattentions") AND **("Acoustic Impedance Tests"[Mesh] OR "Reflex, Acoustic"[Mesh]** OR **"Acoustic Reflex"** OR **"Acoustic Impedance Test"** OR "Impedance Audiometry" OR "Tympanometry" OR "Electroacoustic Impedance Tests" OR "Electroacoustic Impedance Test")
Embase	**("Auditory Perception"** OR "Auditory Perceptions" OR "Auditory Processing" OR "Auditory behavior" OR "Auditory Behaviors" OR "Behavioral Auditory" OR **"Auditory Perceptual Disorders"** OR "Auditory Perceptual Disorder" OR "Auditory Processing Disorder" OR "Auditory Processing Disorders" OR "Psychoacoustical Disorders" OR "Psychoacoustical Disorder" OR "Acoustic Perceptual Disorder" OR "Acoustic Perceptual Disorders" OR "Auditory Comprehension Disorder" OR "Auditory Comprehension Disorders" OR "Auditory Inattention" OR "Auditory Inattentions") AND **("Acoustic Reflex"** OR **"Acoustic Impedance Test"** OR "Impedance Audiometry" OR "Tympanometry" OR "Electroacoustic Impedance Tests" OR "Electroacoustic Impedance Test")
SCOPUS	**("Auditory Perception"** OR "Auditory Perceptions" OR "Auditory Processing" OR "Auditory behavior" OR "Auditory Behaviors" OR "Behavioral Auditory" OR **"Auditory Perceptual Disorders"** OR "Auditory Perceptual Disorder" OR "Auditory Processing Disorder" OR "Auditory Processing Disorders" OR "Psychoacoustical Disorders" OR "Psychoacoustical Disorder" OR "Acoustic Perceptual Disorder" OR "Acoustic Perceptual Disorders" OR "Auditory Comprehension Disorder" OR "Auditory Comprehension Disorders" OR "Auditory Inattention" OR "Auditory Inattentions") AND **("Acoustic Reflex"** OR **"Acoustic Impedance Test"** OR "Impedance Audiometry" OR "Tympanometry" OR "Electroacoustic Impedance Tests" OR "Electroacoustic Impedance Test")
Web of Science	**("Auditory Perception"** OR "Auditory Perceptions" OR "Auditory Processing" OR "Auditory behavior" OR

	"Auditory Behaviors" OR "Behavioral Auditory" OR **"Auditory Perceptual Disorders"** OR "Auditory Perceptual Disorder" OR "Auditory Processing Disorder" OR "Auditory Processing Disorders" OR "Psychoacoustical Disorders" OR "Psychoacoustical Disorder" OR "Acoustic Perceptual Disorder" OR "Acoustic Perceptual Disorders" OR "Auditory Comprehension Disorder" OR "Auditory Comprehension Disorders" OR "Auditory Inattention" OR "Auditory Inattentions") AND **("Acoustic Reflex"** OR **"Acoustic Impedance Test"** OR "Impedance Audiometry" OR "Tympanometry" OR "Electroacoustic Impedance Tests" OR "Electroacoustic Impedance Test")
LILACS	**("Auditory Perception"** OR "Auditory Perceptions" OR "Auditory Processing" OR "Auditory behavior" OR "Auditory Behaviors" OR "Behavioral Auditory" OR **"Auditory Perceptual Disorders"** OR "Auditory Perceptual Disorder" OR "Auditory Processing Disorder" OR "Auditory Processing Disorders" OR "Psychoacoustical Disorders" OR "Psychoacoustical Disorder" OR "Acoustic Perceptual Disorder" OR "Acoustic Perceptual Disorders" OR "Auditory Comprehension Disorder" OR "Auditory Comprehension Disorders" OR "Auditory Inattention" OR "Auditory Inattentions" OR "Percepção Auditiva" OR "Percepções auditivas" OR "Processamento Auditivo" OR "Comportamento auditivo" OR "Comportamentos auditivos" OR "Auditivo Comportamental" OR "Transtornos da Percepção Auditiva" OR "Transtorno da Percepção Acústica" OR "Percepción Auditiva" OR "Percepciones auditivas" OR "Procesamiento Auditivo" OR "Comportamiento auditivo" OR "Comportamientos auditivos" OR "Conductual Auditivo" OR "Trastornos de la Percepción Auditiva" OR "trastorno perceptivo acústico" OR "trastornos de la percepción auditiva") AND **("Acoustic Reflex"** OR **"Acoustic Impedance Test"** OR "Impedance Audiometry" OR "Tympanometry" OR "Electroacoustic Impedance Tests" OR "Electroacoustic Impedance Test" OR "Reflexo Acústico" OR "Testes de Impedância Acústica" OR "Audiometria de Impedância" OR "Teste de Impedância Acústica" OR "Teste de Impedância Eletroacústica" OR "Testes de Impedância Eletroacústica" OR "Timpanometria" OR "Reflejo Acústico" OR "Pruebas de Impedancia Acústica" OR "prueba de impedancia electroacústica" OR "pruebas de impedancia electroacústica")
SCIELO	**("Auditory Perception"** OR "Auditory Perceptions" OR "Auditory Processing" OR "Auditory behavior" OR "Auditory Behaviors" OR "Behavioral Auditory" OR **"Auditory Perceptual Disorders"** OR "Auditory Perceptual Disorder" OR "Auditory Processing Disorder" OR "Auditory Processing Disorders" OR "Psychoacoustical

	Disorders" OR "Psychoacoustical Disorder" OR "Acoustic Perceptual Disorder" OR "Acoustic Perceptual Disorders" OR "Auditory Comprehension Disorder" OR "Auditory Comprehension Disorders" OR "Auditory Inattention" OR "Auditory Inattentions" OR "Percepção Auditiva" OR "Percepções auditivas" OR "Processamento Auditivo" OR "Comportamento auditivo" OR "Comportamentos auditivos" OR "Auditivo Comportamental" OR "Transtornos da Percepção Auditiva" OR "Transtorno da Percepção Acústica" OR "Percepción Auditiva" OR "Percepciones auditivas" OR "Procesamiento Auditivo" OR "Comportamiento auditivo" OR "Comportamientos auditivos" OR "Conductual Auditivo" OR "Trastornos de la Percepción Auditiva" OR "trastorno perceptivo acústico" OR "trastornos de la percepción auditiva") AND **("Acoustic Reflex"** OR **"Acoustic Impedance Test"** OR "Impedance Audiometry" OR "Tympanometry" OR "Electroacoustic Impedance Tests" OR "Electroacoustic Impedance Test" OR "Reflexo Acústico" OR "Testes de Impedância Acústica" OR "Audiometria de Impedância" OR "Teste de Impedância Acústica" OR "Teste de Impedância Eletroacústica" OR "Testes de Impedância Eletroacústica" OR "Timpanometria" OR "Reflejo Acústico" OR "Pruebas de Impedancia Acústica" OR "prueba de impedancia electroacústica" OR "pruebas de impedancia electroacústica")
ProQuest	noft(("Auditory Perception" OR "Auditory Perceptions" OR "Auditory Processing" OR "Auditory behavior" OR "Auditory Behaviors" OR "Behavioral Auditory" OR "Auditory Perceptual Disorders" OR "Auditory Perceptual Disorder" OR "Auditory Processing Disorder" OR "Auditory Processing Disorders" OR "Psychoacoustical Disorders" OR "Psychoacoustical Disorder" OR "Acoustic Perceptual Disorder" OR "Acoustic Perceptual Disorders" OR "Auditory Comprehension Disorder" OR "Auditory Comprehension Disorders" OR "Auditory Inattention" OR "Auditory Inattentions") AND ("Acoustic Reflex" OR "Acoustic Impedance Test" OR "Impedance Audiometry" OR "Tympanometry" OR "Electroacoustic Impedance Tests" OR "Electroacoustic Impedance Test"))
LIVIVO	**("Auditory Perception"** OR "Auditory Perceptions" OR "Auditory Processing" OR "Auditory behavior" OR "Auditory Behaviors" OR "Behavioral Auditory" OR **"Auditory Perceptual Disorders"** OR "Auditory Perceptual Disorder" OR "Auditory Processing Disorder" OR "Auditory Processing Disorders" OR "Psychoacoustical Disorders" OR "Psychoacoustical Disorder" OR "Acoustic Perceptual Disorder" OR "Acoustic Perceptual Disorders" OR "Auditory Comprehension Disorder" OR "Auditory Comprehension Disorders" OR "Auditory Inattention" OR

	"Auditory Inattentions") AND **("Acoustic Reflex"** OR **"Acoustic Impedance Test"** OR "Impedance Audiometry" OR "Tympanometry" OR "Electroacoustic Impedance Tests" OR "Electroacoustic Impedance Test")
Google Scholar	("Auditory Processing Disorder" OR "Processamento Auditivo" OR "Procesamiento Auditivo") AND ("Acoustic Reflex" OR "Reflexo Acústico" OR "Reflejo Acústico")
