# Supplementary material for: Association Between the Alteration of Acoustic Reflex and Central Auditory Processing Disorder in Children and Adolescents: A Systematic Review
Source: Int Arch Otorhinolaryngol. 2026 Oct 23;30(4):1–8. doi: 10.1055/a-2804-5151 (PMC13694147; doi:10.1055/a-2804-5151)
Supplement: Supplementary file 2 [file 10-1055-a-2804-5151-s251986-2.pdf]

Appendix 2. Excluded articles and reason for exclusions (n= 15)

Author, Year	Reason for exclusions
Bartz et al 2015[1]	10
Bertognolli & Buss 2016[2]	9
Burguetti et al 2008[3]	1
Choi et al 2018[4]	2
Deconde 1982[5]	5
Dermott et al 2016[6]	6
Etges et al 2012[7]	3
Franciozi et al 2022[8]	3
Leles et al 2014[9]	8
Lima et al 2021[10]	9
Linares & Carvallo 2004[11]	4
Sant'Anna et al 2015[12]	9
Santos et al 2015[13]	7
Thomas et al 1995[14]	7
Topolska et al 2006[15]	5

1- Sensitized AR (n=1), 2-Sample including Autism Spectrum Disorder (n=1), 3- Did not perform CAP assessment (n=2), 4- Sample included only the presence of AR (n=1), 5- Full article /thesis not found (n=2), 6- Did not compare AR with CAPD (n=1), 7- Sample included adults (n=2), 8- Did not report participants' age (n=1), 9- Conference abstract (n=3), 10 – Performed only one CAP test (n=1).

1 - Bartz DW, Laux CN, Peruch CV, Inês M, Machado MS, Ribas LP. Relação entre os achados do teste masking level difference e do reflexo acústico em crianças com transtorno fonológico. Rev CEFAC. 2015 Set;17(5):1499–508.

2 - Bertagnolli AP, Buss LH. The auditory process and the most impaired auditory skills in children with learning disabilities. Int Arch Otorhinolaryngol. 2016.

3 - Burguetti FAR, Carvallo RMM. Sistema auditivo eferente: efeito no processamento auditivo. Rev Bras Otorrinolaringol. 2008;74(5):737-45. doi: 10.1590/s0034-72992008000500016.

4 - Choi SMR, Kei J, Wilson WJ. Hearing and Auditory Processing Abilities in Primary School Children with Learning Difficulties. Ear Hear. 2019;40(3):700-9. doi: 10.1097/aud.0000000000000652.

5 - Deconde CR. Acoustic Reflex Measurements in Children with Central Auditory Processing Disorders. 1983.

6 - Etges CL, Dos Reis MCP, Menegotto IH, Sleifer P, Soldera CLC. Achados na triagem imitanciométrica e de processamento auditivo em escolares. Rev CEFAC. 2012 Abr 5;14(6):1098–107.

7 - Franciozi C, Borges VMS, Sleifer P. Associação entre ausência do reflexo acústico contralateral e queixa de aprendizagem. Europub J Health Res. 2022;3(4):354-60.

8 - Leles PM, Pacheco SST, Castro MP, Reis ACM, Mathias ÉL, Coelho LMF, Marangoni AC. Relação entre ausência do reflexo do músculo estapédio e presença de distúrbios do processamento auditivo (central). Rev Cefac. 2013;16(2):438-45. doi: 10.1590/s1516-18462013005000040.

9 - Lima MS de. Associação entre reflexo acústico contralateral e queixas de alterações no processamento auditivo central. In: XXXIII Salão de Iniciação Científica da UFRGS. 2021.

10 - Linares AE, Carvallo RMM. Acoustic Reflex Latency in Children with Auditory Processing Disorder. Int Arch Otorhinolaryngol. 2004;8(1):11-8.

11 - McDermott EE, Smart JL, Boiano JA, Bragg LE, Colon TN, Hanson EM, Emanuel DC, Kelly AS. Assessing Auditory Processing Abilities in Typically Developing School-Aged Children. J Am Acad Audiol. 2016 Feb;27(2):72-84. doi: 10.3766/jaaa.14050. PMID: 26905528.

12 - Sant'Anna BS, Dos Santos Abon Zarh L, Dos Nascimento RA, Ribas LP. Performance in MLD tests (masking level difference) and PSI (pediatric speech intelligibility) of children with abnormal acoustic reflex. Int Arch Otorhinolaryngol. 2015.

13 - Santos TS, Mancini PC, Sancio LP, Castro AR, Labanca L, Resende LM. Achados da avaliação comportamental e eletrofisiológica do processamento auditivo. Audiol Commun Res. 2015;20(3):225-32. doi: 10.1590/2317-6431-2015-1589.

14 - Thomas WG, McMurry G, Pillsbury HC. Acoustic reflex abnormalities in behaviorally disturbed and language delayed children. Laryngoscope. 1985 Jul;95(7 Pt 1):811-7. PMID: 4010421.

15 - Topolska MM, Hassmann-Poznańska E. Analysis of usefulness of central auditory processes tests in children. Otolaryngol Pol. 2015;60(3).
